# Activation of Nrf2 and FXR via Natural Compounds in Liver Inflammatory Disease

**DOI:** 10.3390/ijms252011213

**Published:** 2024-10-18

**Authors:** Marta Belka, Aleksandra Gostyńska-Stawna, Maciej Stawny, Violetta Krajka-Kuźniak

**Affiliations:** 1 Department of Pharmaceutical Biochemistry, Poznan University of Medical Sciences, Rokietnicka 3, 60-806 Poznan, Poland; mbelka@ump.edu.pl; 2 Doctoral School, Poznan University of Medical Sciences, Bukowska 70, 60-812 Poznan, Poland; 3 Department of Pharmaceutical Chemistry, Poznan University of Medical Sciences, Rokietnicka 3, 60-806 Poznan, Poland; agostynska@ump.edu.pl (A.G.-S.); mstawny@ump.edu.pl (M.S.)

**Keywords:** FXR, Nrf2, liver inflammation, MAFLD

## Abstract

Liver inflammation is frequently linked to oxidative stress and dysregulation of bile acid and fatty acid metabolism. This review focuses on the farnesoid X receptor (FXR), a critical regulator of bile acid homeostasis, and its interaction with the nuclear factor erythroid 2-related factor 2 (Nrf2), a key modulator of cellular defense against oxidative stress. The review explores the interplay between FXR and Nrf2 in liver inflammatory diseases, highlighting the potential therapeutic effects of natural FXR agonists. Specifically, compounds such as auraptene, cafestol, curcumin, fargesone A, hesperidin, lycopene, oleanolic acid, resveratrol, rutin, ursolic acid, and withaferin A are reviewed for their ability to modulate both the FXR and Nrf2 pathways. This article discusses their potential to alleviate liver inflammation, oxidative stress, and damage in diseases such as metabolic-associated fatty liver disease (MAFLD), cholestatic liver injury, and viral hepatitis. In addition, we address the molecular mechanisms driving liver inflammation, including oxidative stress, immune responses, and bile acid accumulation, while also summarizing relevant experimental models. This review emphasizes the promising therapeutic potential of targeting both the Nrf2 and FXR pathways using natural compounds, paving the way for future treatments for liver diseases. Finally, the limitations of the clinical application were indicated, and further research directions were proposed.

## 1. Introduction

Inflammatory liver diseases represent a significant global health challenge, impacting millions of people worldwide. The mechanism of liver inflammation is complex and may be caused by metabolic disorders, autoimmune reactions, and genetic diseases. Signaling pathways, such as the Nuclear factor erythroid 2-related factor 2/Antioxidant Response Element (Nrf2/ARE), play pivotal roles in the pathogenesis of liver disease, with their activation occurring in response to inflammation and oxidative stress [1]. The farnesoid X receptor (FXR), a key regulator of bile acids (BAs) metabolism, is essential for maintaining hepatic homeostasis [2]. Recent studies indicate that natural FXR agonists can modulate the Nrf2 pathway, thereby reducing liver inflammation [3]. This discovery presents a promising therapeutic strategy for treating inflammatory liver diseases, paving the way for pharmacological and natural interventions. This paper focuses on the impact of modulating the Nrf2 pathway through natural FXR agonists on liver inflammation reduction. It also reviews recent advancements in research on Nrf2 activators in diseases such as metabolic-associated fatty liver disease (MAFLD), cholestatic liver injury, and hepatitis, aiming to guide future therapeutic approaches for hepatic inflammatory diseases.

We provide research findings from papers that were based on searches of the databases PubMed, Scopus, Web of Science, and Google Scholar using the following terms: “natural compounds”, “FXR”, “Nrf2”, and “liver”.

## 2. Hepatic Inflammatory Diseases 

The most prevalent types of inflammatory liver diseases include viral hepatitis (caused by hepatitis B and C viruses), autoimmune hepatitis, metabolic-associated fatty liver disease (MAFLD), and nonalcoholic steatohepatitis (NASH). Viral hepatitis remains a major global health concern, with chronic hepatitis B (HBV) affecting approximately 296 million people worldwide, and hepatitis C (HCV) infecting around 58 million [4]. Autoimmune hepatitis, though less common, has an estimated incidence of 1–2 cases per 100,000 people annually in regions like Europe and North America [5]. NASH, part of the broader spectrum of metabolic-associated fatty liver disease (MAFLD), is increasingly prevalent, affecting between 1.5% and 6.5% of the global population, driven mainly by rising rates of obesity and metabolic syndrome [6].

It is important to remember that the causes of these diseases are diverse. Viral hepatitis is mainly caused by viruses A, B, and C, which are primarily transmitted through blood and body fluids, leading to chronic inflammation and potential liver damage [7]. Autoimmune hepatitis results from the immune system mistakenly attacking liver cells, possibly triggered by genetic predispositions and environmental factors [8]. MAFLD is a benign condition, and fat accumulates in the liver that can cause inflammation but not progressive damage or complications. NASH is a malignant condition that develops due to fat accumulation in the liver, often associated with insulin resistance, obesity, and poor dietary habits [9]. 

According to the consensus on liver disease terminology published in 2020 in Gastroenterology [10], we used the term “metabolic-associated fatty liver disease” (MAFLD) to express NAFLD and NASH throughout the remainder of this article.

As mentioned earlier, liver inflammation involves intricate mechanisms triggered by various factors, including infections, toxins, autoimmune reactions, and metabolic disorders. Inflammatory processes in the liver involve various immune cells, including T and B lymphocytes, eosinophils, neutrophils, and natural killer (NK) cells, which interact with Kupffer cells and other hepatic cells to produce inflammatory cytokines and modulate cell behavior, potentially leading to liver fibrosis and other complications. T lymphocytes, particularly CD4+ and CD8+ T cells, contribute to liver inflammation by secreting pro-inflammatory cytokines like interferon-gamma (IFN-γ) and tumor necrosis factor-alpha (TNF-α), which enhance Kupffer cell activation. B lymphocytes, primarily known for antibody production, can also contribute to cytokine release and antigen presentation in the liver, promoting inflammation. Eosinophils and neutrophils, as part of the innate immune response, infiltrate the liver and release cytotoxic molecules and pro-inflammatory cytokines, such as interleukin-1β (IL-1β) and TNF-α, amplifying liver damage [11]. 

Monocytes and macrophages contribute to inflammation by producing large amounts of nitric oxide (NO) and inflammatory cytokines, such as TNF-α, directly stimulating stellate cell collagen synthesis. Kupffer cells, resident macrophages in the liver sinusoids, play a significant role in hepatic injury, including endotoxin-mediated damage. These cells express death ligands such as TNF-α, TNF-related apoptosis-inducing ligand (TRAIL), and Fas ligand, which are thought to further stimulate death receptor-mediated apoptosis in the liver. Emerging evidence suggests that this type of apoptosis may contribute to liver inflammation and fibrosis. Fas agonists can induce chemokine expression, promote neutrophil infiltration, and stimulate hepatic fibrogenesis [12].

NK cells, another component of the innate immune system, interact with Kupffer cells and hepatocytes, secreting IFN-γ to promote inflammation and liver cell death. These immune cells’ collective activity and interactions with Kupffer cells drive the production of inflammatory cytokines, perpetuating liver injury and fibrosis in conditions like hepatitis, MAFLD, and autoimmune liver diseases [13].

Biomarkers of liver inflammatory diseases are elevated levels of enzymes like alanine aminotransferase (ALT), aspartate aminotransferase (AST), lactate dehydrogenase (LDH), alkaline phosphatase (ALP), and gamma-glutamyl transferase (γ-GT). Another marker is bilirubin, a yellow pigment resulting from the breakdown of red blood cells. Monitoring ALT, AST, LDH, ALP, γ-GT, and bilirubin levels are crucial for diagnosing and assessing the severity of liver diseases [14,15].

Oxidative stress occurs when there is an imbalance between the production of reactive oxygen species (ROS) and the body’s ability to detoxify these harmful compounds through antioxidant mechanisms. In the liver, oxidative stress can damage hepatocytes, contributing to inflammation, fibrosis, and the progression of liver diseases such as hepatitis, MAFLD, and liver cirrhosis. The inflammatory process induces oxidative stress, reducing the cellular antioxidant capacity. Overproduction of free radicals leads to the permanent impairment of cell membrane fatty acids and proteins. Chronic inflammation is considered a major precursor to cancer development. ROS and inflammatory cytokines like TNF-α activate the transcription factor nuclear factor kappa-B (NF-κB) through phosphorylation and subsequent proteasomal degradation of the connected IκB subunit. Released NF-κB then migrates to the nucleus and activates the transcription of specific genes involved in cell proliferation, apoptosis, and carcinogenesis. It also induces the production of proinflammatory cytokines, further amplifying inflammatory responses [16].

Oxidative stress is also a key factor in activating the Nrf2/ARE signaling pathway. Nuclear factor erythroid 2-related factor 2 (Nrf2) is a transcription factor that plays a vital role in cellular defense against oxidative stress. Its activation by ROS is a critical mechanism in the cellular response to oxidative damage. Nrf2 regulates the basal and inducible expression of numerous cytoprotective genes, playing a pivotal role in protecting against oxidative damage. Furthermore, studies indicate that NF-κB can activate Nrf2 expression at the transcriptional level due to several functional binding sites in the promoter region of the NFE2L2 gene, thereby inducing a positive feedback loop [17].

## 3. Liver Inflammation Research Models

Given the crucial roles of oxidative stress and inflammation in the pathophysiological changes associated with liver diseases, Nrf2 and NF-κB interact to regulate antioxidant and inflammatory responses. Understanding these mechanisms is essential for developing therapeutic strategies to manage and treat liver inflammation and its associated conditions. To explore these interactions and their therapeutic potential, various experimental models have been developed to study liver inflammation and oxidative stress. Both in vitro and in vivo studies utilize a range of cell lines and animal models to replicate the conditions observed in liver diseases, particularly MAFLD. Different cell lines are commonly used in in vitro studies of liver inflammation and oxidative stress. For instance, cell cultures such as human liver cancer cell line HepG2, human hepatocellular carcinoma cell line HepaRG, monocytic cell line THP-1, human hepatic stellate cell line LX-2, and primary hepatocytes are employed in MAFLD research [18]. In vivo experiments in this field are typically conducted on mice and rats. The methods used to induce liver damage in these models can be grouped into chemical, dietary, surgical, transgenic, and immunological approaches [19]. Commonly used chemical agents include ethanol, carbon tetrachloride (CCl_4_), α-naphthyl isothiocyanate (ANIT), and di-(2-ethylhexyl) phthalate (DEHP). Ethanol promotes the overproduction of ROS and disrupts lipid metabolism in the liver, resulting in ROS-mediated liver injury [19]. A well-known example of a hepatotoxic chemical is CCl_4_, which cytochrome P450 metabolizes into the reactive trichloromethyl radical (·CCl_3_). This metabolite induces oxidative stress and damages mitochondria by binding to proteins, DNA, and lipids [19]. ANIT acts as an indirect liver toxin, affecting intrahepatic bile duct epithelial cells and causing high concentrations of bile acids to be released into the liver, leading to liver damage. This model is widely used to investigate cholestatic liver diseases, as it significantly increases total and direct bilirubin levels [20,21]. Exposure to DEHP triggers oxidative stress and inflammatory reactions that exacerbate liver damage while also causing fat accumulation in the liver [22,23]. Other substances used in liver inflammation models include thioacetamide (TAA), diethylnitrosamine (DEN), dimethylnitrosamine (DMN), and acetaminophen (APAP) [20], among other liver toxins. Dietary induction methods include the methionine-choline-deficient diet (MCD), high-fat diet (HFD), choline-deficient l-amino acid-defined (CDAA) diet, and the choline-deficient, l-amino acid-defined, high-fat diet (CDAHFD). HFD is commonly used to induce metabolic syndrome, hepatic steatosis, and MAFLD in experimental animals. HFD-fed models effectively mimic the metabolic abnormalities of MAFLD and other oxidative stress and inflammation-related conditions [20,24]. Bile duct ligation (BDL) is the most commonly performed surgical procedure and the most established experimental model for cholestasis. The BDL model demonstrates hepatic damage characterized by histological alterations, elevated serum biochemistry, fibrosis, and inflammation. BDL causes severe inflammation, oxidative stress, and fibrosis in the liver [19].

## 4. Canonical and Non-Canonical Mechanism of Nrf2 Activation 

**Figure 1:**
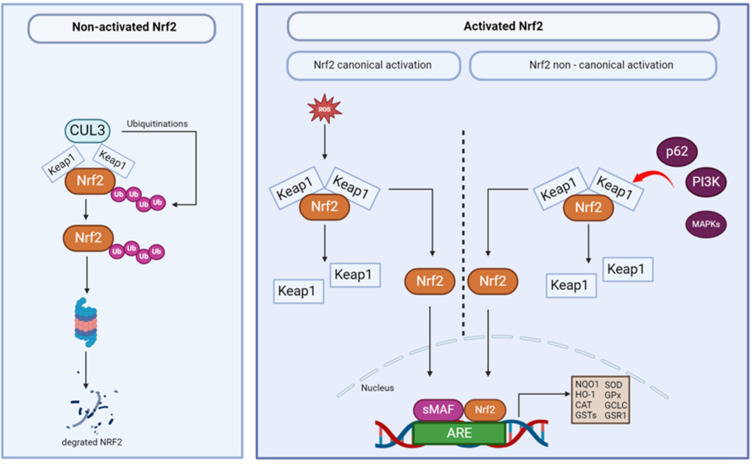
Canonical and non-canonical mechanisms of Nrf2 activation. Abbreviations: ARE, Antioxidant Response Element; CAT, catalase; Cul3, Cullin 3; GCLC, glutamate-cysteine ligase catalytic subunit; GPx, glutathione peroxidase; GSTs, glutathione S-transferases; GSR1, glutathione reductase 1; HO-1, heme oxygenase-1; Keap1, Kelch-like ECH-associated protein 1; MAPKs, mitogen-activated protein kinases; NQO1, NAD(P)H oxidoreductase 1; Nrf2, Nuclear factor erythroid 2-related factor 2; p62, Sequestosome 1; PI3K, phosphatidylinositol 3-kinase; ROS, reactive oxygen species; sMAF, small musculoaponeurotic fibrosarcoma oncogene homolog; SOD, superoxide dismutase; Ub, ubiquitination. Created with BioRender.com.

Nuclear factor erythroid 2-related factor 2 (Nrf2) is a transcription factor crucial in cellular defense against oxidative and electrophilic stress. Nrf2 plays a pivotal role in protecting the liver from injury by regulating the Nrf2/ARE pathway, enhancing the expression of proteins that detoxify reactive molecules in various cell compartments, thus promoting cellular adaptation and survival. Moreover, in the context of liver inflammation, activation of Nrf2 can mitigate oxidative stress, help to protect hepatocytes from injury, and reduce inflammation, making Nrf2 a potential therapeutic target in liver diseases. There are two types of Nrf2 activation: canonical and non-canonical (Figure 1). 

The canonical pathway of Nrf2 activation refers to the regulatory mechanism of this protein by its inhibitor, Keap1 (Kelch-like ECH-associated protein 1). Keap1 functions as a substrate adaptor for the Cullin 3 (Cul3)-based E3 ubiquitin ligase complex, promoting the ubiquitination and subsequent degradation of Nrf2 by the proteasome. Under oxidative stress or electrophilic insults, Keap1 undergoes conformational changes, leading to the release and subsequent stabilization of Nrf2. Stabilized Nrf2 translocates into the nucleus, forming a heterodimer with small Maf proteins (musculoaponeurotic fibrosarcoma oncogene homolog). The binding of Nrf2 to Antioxidant Response Elements (AREs) initiates the transcription of various genes involved in cellular defense mechanisms against oxidative stress. These target genes include phase II detoxification enzymes such as NAD(P)H oxidoreductase 1 (NQO1), heme oxygenase-1 (HO-1), and antioxidant proteins such as catalase (CAT), superoxide dismutase (SOD) glutathione S-transferases (GSTs), glutathione peroxidase (GPx), glutamate-cysteine ligase catalytic subunit (GCLC), and glutathione reductase 1 (GSR1) [25].

The non-canonical Nrf2 activation pathway operates independently of the traditional Keap1-mediated mechanism and involves alternative strategies for triggering Nrf2 activity. p62 (also known as SQSTM1, Sequestosome 1) is a crucial adaptor protein in this process, participating in various cellular functions, such as autophagy, protein degradation, and stress response. In the non-canonical pathway, p62 contains a specific STGE motif within its ubiquitin-associated (UBA) domain, allowing it to bind to Keap1 competitively, inhibiting Keap1’s ability to ubiquitinate and degrade Nrf2. As a result, Nrf2 is released from the Keap1-Nrf2 complex, allowing it to translocate into the nucleus, where it binds to ARE and initiates the transcription of protective genes [26].

This pathway is further modulated by protein kinases, such as phosphatidylinositol 3-kinase (PI3K) and mitogen-activated protein kinases (MAPKs), as well as by external stimuli like growth factors, cytokines, and hormones. Once activated, Nrf2 forms heterodimers with small Maf proteins or other transcription factors, depending on the specific context, and binds to AREs in the promoter regions of target genes. These target genes often overlap with those regulated by the canonical pathway, contributing to cellular defense against oxidative stress and the maintenance of redox balance [27,28].

As mentioned earlier, the Keap1-Nrf2-ARE pathway is activated in response to oxidative or electrophilic stress, regulating the expression of proteins involved in detoxifying reactive molecules in the cytosol and the mitochondria and endoplasmic reticulum (ER). This response protects cells from damage and promotes survival. Cellular adaptation is also supported by the unfolded protein response (UPR), which restores ER homeostasis, and the autophagy–lysosomal pathway, which degrades dysfunctional proteins and organelles. Evidence suggests a connection between hepatic steatosis and ER stress, as several lipogenic pathways are in the ER. UPR disruption can lead to ER stress-induced steatosis, and protein kinase RNA-like endoplasmic reticulum kinase (Perk)-dependent phosphorylation during UPR may cause Nrf2 nuclear translocation, increasing the transcription of its target genes. Research has shown that Nrf2 is essential for mitochondrial integrity and energy production, as mice deficient in Nrf2 are more susceptible to liver damage caused by oxidative stress, chemical toxins, and excessive iron accumulation [29,30].

## 5. Farnesoid X Receptor

Farnesoid X receptor (FXR), a nuclear receptor predominantly expressed in the liver and intestines, is crucial for maintaining BA homeostasis. BAs, which are essential for fat digestion and absorption, can cause oxidative stress and liver injury when accumulated excessively. FXR activation regulates genes involved in BA synthesis, transport, and detoxification. It decreases BA production by inhibiting cholesterol 7α-hydroxylase (CYP7A1) and enhances BA excretion. Additionally, FXR activation exerts anti-inflammatory effects by reducing pro-inflammatory cytokines and repressing the NF-κB signaling pathway, which drives liver inflammation.

BAs, derived from cholesterol, are vital for digestion and act as signaling molecules that regulate various metabolic processes. Usually, the FXR tightly controls BA levels to prevent their accumulation, thereby reducing oxidative stress and inflammation. Dysregulation of BA metabolism during liver diseases can exacerbate oxidative stress, damaging hepatocyte membranes, releasing pro-inflammatory cytokines, and promoting liver inflammation. The FXR helps mitigate these effects by controlling BA levels and reducing oxidative stress. The FXR, part of the broader family of metabolic nuclear receptors, is mainly found in the liver, intestine, kidney, adrenals, and heart [31]. The FXR family includes FXRα and FXRβ, with FXRβ being a human pseudogene. The FXRα gene codes for four isoforms: FXRα1, FXRα2, FXRα3, and FXRα4. While FXRα1 and FXRα2 manifest the highest expression in the liver, FXRα3 and FXRα4 occur most often in the intestine and kidney [32]. FXR follows the typical nuclear receptor structure with a ligand-independent activation domain (AF1), a core DNA-binding domain (DBD), a hinge region, a C-terminal ligand-binding domain (LBD), and a ligand-dependent activation function domain (AF2) [33]. Upon ligand binding to the LBD, FXR activates and reveals docking sites for transcriptional cofactors like PGC-1α, P300, and Sirtuin 1 (SIRT1). Activated FXR and these cofactors bind to FXR response elements (FXREs) on DNA alone or as a heterodimer with retinoid X receptor (RXR) [34]. This binding regulates the transcription of genes involved in BA biosynthesis, transport, and metabolism, which is essential for maintaining appropriate intracellular Ba levels. The FXR protects against excessive BA accumulation by inhibiting cholesterol 7 alpha-hydroxylase (CYP7A1), sterol 12-alpha-hydroxylase (CYP8B1), and sterol 27-hydroxylase (CYP27A1) [35], reducing intrahepatic BA synthesis. It also enhances UDP-glucuronyl transferase 1A1 (UGT1A1) activity for BA metabolism, increases bile salt export pump (BSEP) and organic solute transporter beta (OSTß) expression to facilitate BA secretion, and decreases Na^+^-taurocholate transporting polypeptide (NTCP) and organic anion transporter polypeptides (OATP) activity to impede BA reabsorption [35]. Additionally, FXR activation induces a small heterodimer partner (SHP), which represses NTCP transcription and regulates phase II metabolism enzymes like sulfotransferases 2A1 (SULT2A1). FXR also modulates fatty acid metabolism in the liver by inhibiting sterol regulatory element-binding transcription factor 1c (SREBP-1c) [36] and stearoyl-CoA desaturase (SCD1) [37], while activating AMP-activated protein kinase (AMPK) [38].

## 6. Interconnection between Nrf2 and FXR in Liver Inflammation 

**Figure 2:**
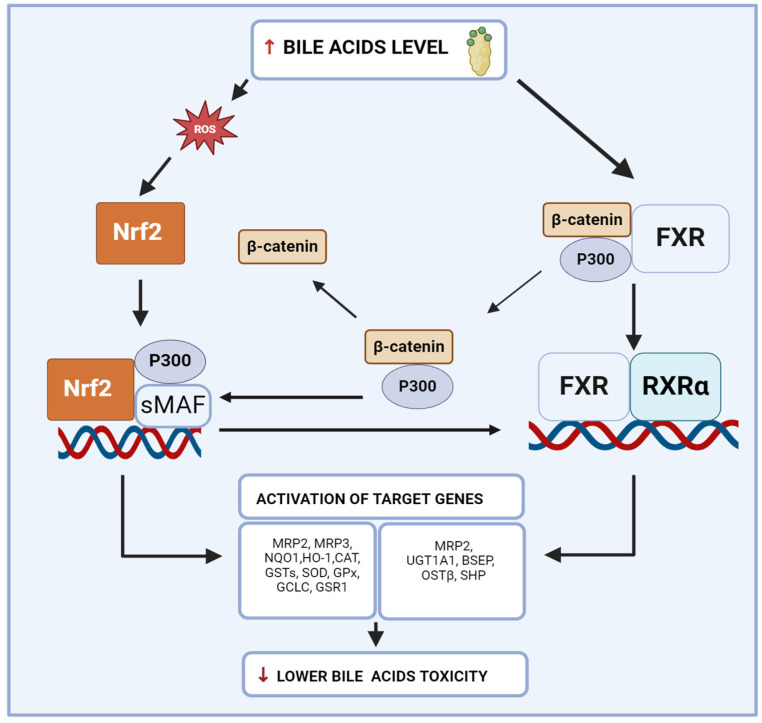
Interconnection between Nrf2 and FXR in inflammation. ↑/↓ increase/decrease. Abbreviations: BSEP, bile salt export pump; CAT, catalase; FXR, farnesoid X receptor; GCLC, glutamate-cysteine ligase catalytic subunit; GPx, glutathione peroxidase; GSTs, glutathione S-transferases; GSR1, glutathione reductase 1; HO-1, heme oxygenase-1; MRP2, multidrug resistance-associated proteins 2; MRP3, multidrug resistance-associated proteins 3; NQO1, NAD(P)H oxidoreductase 1; Nrf2, nuclear factor erythroid 2-related factor 2; OSTß, organic solute transporter beta; P300, P300 protein; β-catenin, beta-catenin; ROS, reactive oxygen species; RXRα, retinoid X receptor; SHP, small heterodimer partner, sMAF, small musculoaponeurotic fibrosarcoma oncogene homolog; SOD, superoxide dismutase; UGT1A1, UDP-glucuronyl transferase 1A1. Created with BioRender.com.

Nrf2 and FXR are interconnected in their roles in managing oxidative stress and inflammation in the liver (Figure 2). While Nrf2 primarily focuses on detoxifying ROS and reducing oxidative damage, the FXR maintains BA homeostasis and prevents BA-induced oxidative stress. The coordinated regulation by Nrf2 and the FXR is essential for protecting the liver from inflammation and the progression of chronic liver diseases.

As mentioned earlier, Nrf2 activation in the liver enhances lipid profiles and reduces BA accumulation. Studies in mice have shown that Nrf2 activation lowers BA concentrations in the liver, likely due to decreased hepatic BA synthesis [39]. Since the FXR plays a crucial role in BA metabolism, exploring the interplay between Nrf2 and FXR pathways is essential. Research indicates that proteins such as P300 and β-catenin mediate this interaction [40]. β-catenin contributes to cholestasis in inflammatory conditions and inhibiting it can reduce BA accumulation by activating FXR. Lowering β-catenin levels increases oxidative stress and promotes Nrf2 activation. In mice with liver-specific β-catenin knockout, Nrf2 activation was enhanced, with increased Nrf2 acetylation, Nrf2 binding to P300, and transcription of Nrf2 target genes in a MAFLD model. Moreover application of GW4064 (FXR activator) also decreased β-catenin’s binding to P300, weakening β-catenin’s stability with the FXR and enhancing the FXR’s interaction with Retinoid X Receptor (RXR) [40].

The activated FXR facilitates transcription of downstream genes and reduces FXR binding to ß-catenin, which increases P300 binding to Nrf2. These findings suggest a close relationship among β-catenin, P300, Nrf2, and FXR signaling pathways, vital for maintaining BA homeostasis [40,41]. Additional studies support these connections. For instance, Yan’s research on Liquiritin (LQ), a compound from *Glycyrrhiza glabra*, found that LQ upregulated the FXR and Nrf2, suggesting that the FXR and SIRT1/Nrf2 pathways are targets of LQ’s protective effects [42]. The FXR and Nrf2 influence the expression of multidrug resistance-associated proteins (MRPs). MRP2, located on the canalicular membrane of hepatocytes, transports organic anions and conjugates like bilirubin glucuronides. Its expression can be downregulated in inflammatory liver diseases, leading to cholestasis and jaundice. Conversely, MRP3 and MRP4 on the basolateral membrane transport BAs, conjugated bilirubin, and other organic anions into the blood. Upregulation of MRP3 and MRP4 can serve as a compensatory mechanism during cholestasis to reduce intracellular BA accumulation. The FXR induces MRP2 expression to facilitate BA excretion and protect against cholestasis, while Nrf2 activation upregulates MRPs such as MRP1, MRP2, and MRP3, enhancing cellular detoxification and protecting against oxidative and chemical stress [43].

High concentrations of BAs can damage hepatocyte membranes, releasing pro-inflammatory cytokines and promoting liver inflammation. This can lead to necrosis, apoptosis, and cell death [44]. BA-induced oxidative stress is linked to ROS generated through changes in mitochondrial respiration. Additionally, BAs initiate an inflammatory response, attracting neutrophils and macrophages to the liver, where they release ROS. Inhibiting neutrophil function has been shown to reduce oxidative stress and liver injury, indicating that neutrophils are a significant source of damaging ROS in vivo [45].

Additionally, research has indicated that oxidative stress occurs in the livers of people suffering from cholestasis. Furthermore, inhibition of ROS during cholestasis protects against liver fibrosis [45]. Elevation of serum ROS levels was strongly linked with cholestasis [46], suggesting that ROS can be used as a disease marker. Numerous investigations have demonstrated that oxidative stress occurs in BDL animal cholestasis models [45].

In summary, oxidative stress plays a significant role in liver inflammation, and the activation of Nrf2 and the FXR provides protective mechanisms against such damage. Nrf2 enhances the liver’s antioxidant defenses, while the FXR ensures BA homeostasis, both of which are crucial for preventing liver disease progression.

## 7. Natural Agonists of FXR and Modulators of Nrf2 

The natural agonists of the FXR are BAs, with chenodeoxycholic acid (CDCA) being a high-affinity ligand. Other compounds such as lithocholic acid (LCA), deoxycholic acid (DCA), oxysterol-22(R)-hydroxy cholesterol, androsterone, and polyunsaturated fatty acids like arachidonic acid and docosahexaenoic acid act as weaker natural ligands [47,48]. Obeticholic acid (OCA), a semi-synthetic FXR agonist, exhibits more significant FXR agonistic activity than CDCA. OCA has been investigated for treating conditions like alcoholic hepatitis and nonalcoholic steatohepatitis (NASH) [48]. However, due to adverse side effects, its use has been discontinued, prompting the search for alternative FXR agonists for therapeutic purposes. 

Here, we have outlined and summarized in Table 1 some natural compounds with potential impact on the FXR and Nrf2. 

### 7.1. Auraptene

Auraptene is a natural compound found abundantly in the *Rutaceae* and *Umbelliferae* plant families. This agent shows anti-inflammatory, antioxidant, antidiabetic, antihypertensive, anticancer, and neuroprotective properties [70]. Gao et al. conducted in silico studies to demonstrate that auraptene acts as a ligand for the FXR. To validate this hypothesis further, they performed in vitro and in vivo studies using mouse primary cultured hepatocytes and mice. Their research revealed that auraptene functions as a natural ligand for the FXR, inhibiting the transcription of genes regulated by the FXR, such as CYP7A1, CYP8B1, and NTCP. This inhibition leads to hepatic protection and the suppression of inflammation by blocking the NF-κB pathway [49]. In another study, HepG2 liver cancer cells were transfected with an ARE reporter plasmid and a green fluorescent protein (GFP) reporter and then treated with auraptene, and as a result, auraptene increased GFP fluorescence and GST activity by activating the Nrf2/ARE pathway [50].

### 7.2. Cafestol

Cafestol, a diterpene found in coffee beans from the *Coffea* plant, exhibits various beneficial effects, including anti-inflammatory, hepatoprotective, anticancer, antidiabetic, and anti-osteoclastogenesis activities [71]. It is recognized as a potent FXR agonist. In mice, cafestol has been shown to activate the FXR in the liver, suppressing CYP7A1, CYP8B1, and NTCP expression and reducing BA synthesis. Additionally, cafestol can directly influence the expression of genes involved in cholesterol metabolism by activating the nuclear receptors, the FXR and pregnane X receptor (PXR), with PXR activity being specific to the intestine [51]. The antioxidant properties of cafestol were demonstrated in studies using mouse models with CCl_4_-induced liver damage, where cafestol treatment enhanced GST activities and protected against liver injury [52].

### 7.3. Curcumin

Curcumin extracted from the dried rhizome of *Curcuma longa* exerts hepatoprotective effects by inhibiting lipid synthesis. Multiple studies have shown that curcumin can inhibit SREBP-1c and induce the expression of cytochrome P450 3A (CYP3A) and cytochrome P450 7A1 (CYP7A1) while also stimulating Nrf2 expression [72,73,74]. Additionally, curcumin has been found to modulate the Nrf2/FXR pathway, contributing to improvements in MAFLD [53]. Similar findings were reported by Lu et al., where curcumin inhibited ethanol-induced intracellular lipid accumulation in rats, mediated by the Nrf2 and FXR pathways [3].

### 7.4. Fargesone A

Fargesone A is a natural compound derived from *Magnolia fargesii*, which is traditionally used in Chinese medicine. Due to the challenges of obtaining it through natural extraction, researchers have developed a biomimetic total synthesis of fargesone A. While it exhibits slightly reduced FXR activation compared to OCA, molecular docking studies have shown that fargesone A effectively binds to the hydrophobic pocket of the FXR ligand-binding domain. It demonstrates significant potency and selectivity in activating the FXR over other nuclear receptors. Activation of the FXR by fargesone A has been confirmed to upregulate SHP and BSEP mRNA levels while downregulating CYP7A1 and CYP8B1 mRNA levels [54].

### 7.5. Hesperidin

Hesperidin is a flavanone glycoside found abundantly in lemons (*Citrus limon*), sweet oranges (*Citrus sinensis*), bitter oranges (*Citrus aurantium*), and citron (*Citrus medica*) [75]. Its hepatoprotective effects against cholestasis and hepatotoxicity have been evaluated in mice and both normal and FXR-suppressed HepaRG cells. Hesperidin protects against ANIT-induced cholestatic liver injury by activating the FXR, which promotes BA secretion from the liver and inhibits BA uptake and synthesis. It enhances BA excretion into the feces and reduces hepatic accumulation by regulating FXR target genes such as BSEP, MRP2, and NTCP. Also, hesperidin inhibits BA synthesis enzymes, including CYP27A1 and CYP7A1. Molecular docking studies predict that hesperidin binds to the FXR, and its ability to boost FXR expression and activity is reversed by FXR silencing [55].

In a study by Li et al., hesperidin’s anti-inflammatory and antioxidant properties were examined in an MAFLD model. In HepG2 cells induced with oleic acid (OA), hesperidin increased the levels of antioxidant enzymes such as SOD, GPx, GCLC, and HO-1 by activating the PI3K/Protein kinase B (AKT)/Nrf2 signaling pathway, thus mitigating excessive ROS production. In rats with MAFLD, hesperidin improved lipid profiles, steatosis, inflammation, fibrosis, and liver function [56].

### 7.6. Lycopene

Lycopene is a lipophilic carotenoid extracted from tomatoes (*Solanum lycopersicum*) and red vegetables. In one study, rats fed a high-fat diet and supplemented with tomato juice showed upregulation of the mRNA for the nuclear receptor subfamily 1 group H member (4NR1H4), which encodes the FXR, and an improvement in their lipid profiles [76]. In studies where liver injury was induced using chlorpyrifos and aflatoxin B1 [57,58], lycopene effectively demonstrated its antioxidant properties. Higher levels of Nrf2 protein, as well as increased SOD and CAT activities, were measured. This resulted in lower serum ALT and AST levels, confirming lycopene’s liver-protective properties after toxin administration.

### 7.7. Oleanolic Acid

Oleanolic acid is a pentacyclic triterpenoid found in *Olea europaea* and *Rosa woodsia* plants. It is well known for its antioxidant, anti-inflammatory, antibacterial, antiviral, antihypertensive, anticancer, and hepatoprotective effects [77]. An interesting study was conducted by Liu et al. They tested the influence of oleanolic acid on rats with ANIT-induced liver injury by silencing Nrf2 or FXR genes. The results indicated that oleanolic acid restored the liver damage induced by ANIT and showed a connection between FXR and Nrf2 pathways [40]. 

### 7.8. Resveratrol

Resveratrol, a non-flavonoid phenol, is found in grape peels (*Vitis vinifera*), blueberries (*Vaccinium myrtillus*), raspberries (*Rubus idaeus*), peanuts (*Arachis hypogaea*), and red wine. It has been shown to improve glucose metabolism, enhance lipid profiles, and reduce liver fibrosis and steatosis [78]. A study by Hajighasem et al. investigated the effects of resveratrol on the expression of FXR, liver X receptor (LXR), and SIRT1 genes in the liver of elderly rats with MAFLD. The results demonstrated that resveratrol, either alone or in combination with exercise, significantly improved the expression of SIRT1, LXR, and FXR in hepatic tissue. Additionally, it led to significant reductions in the levels of AST, ALT, and ALP enzymes, as well as apoptotic cells [59].

Resveratrol has also shown protective effects against ANIT-induced cholestasis through FXR regulation. It modulates BA-related genes via the FXR pathway and antagonizes NF-κB activity. Compared to 6α-ethyl chenodeoxycholic acid (6ECDCA), an FXR agonist, resveratrol exhibited similar inhibitory effects on cyclooxygenase-2 (COX-2), iNOS, and TNF-α expression. It was found to reduce the mRNA levels of CYP7A1, CYP8B1, and CYP27A1, which FXR negatively regulates, and to induce the expression of BA transporters such as BSEP and OSTß, thus reducing BAs accumulation in the liver [60]. In studies on resveratrol’s impact on the epigenetic regulation of the Nrf2-Keap1 pathway in MAFLD models, this compound was shown to significantly reduce Keap1 mRNA and protein levels while increasing the methylation status of the Nrf2 promoter. Moreover, it upregulated the expression of Nrf2 target genes, including HO-1, NQO1, CAT, and superoxide dismutase 2 (SOD2), and downregulated genes related to lipogenesis, such as SREBP-1c and fatty acid synthase (FAS). These findings indicate that resveratrol mitigates oxidative stress and lipid accumulation through epigenetic modifications of Nrf2 [61].

### 7.9. Rutin

Rutin is a flavonol found abundantly in passionflower, buckwheat, and tea plants, and it is named after *Ruta graveolens*. Rutin possesses various beneficial properties, including antioxidant, cytoprotective, vasoprotective, anticarcinogenic, hepatoprotective, neuroprotective, and cardioprotective activities [79].

Rutin demonstrates a strong binding affinity for the FXR. In docking studies, rutin bound to the FXR with a higher binding energy (−22.38 kcal/mol) compared to 3-deoxy CDCA (−15.36 kcal/mol) [80,81]. In rat models with BDL-induced liver injury, rutin improved serum biomarkers such as AST, ALT, total bilirubin, TG, and total cholesterol. The study showed that rutin’s inhibition of BDL-induced cholangiocyte and inflammatory cell activation correlated with the downregulation of NF-κB and transforming growth factor beta (TGF-β)/Smad activities, likely through interference with extracellular signal-regulated kinase (ERK) activation and/or enhancement of Nrf2, HO-1, and AMPK activities. Rutin also reduces BDL-induced oxidative stress by attenuating liver glutathione (GSH) depletion [62]. Similar findings were observed in studies of rutin’s effects on diabetic MAFLD, both in vitro and in vivo. Rutin increased the levels of antioxidant proteins such as Nrf2, HO-1, and NQO1 while inhibiting lipid metabolism-related proteins like FAS, SREBP1, and SCD1. This resulted in improved lipid metabolism and reduced dysfunction [63].

### 7.10. Ursolic Acid

Triterpenoid ursolic acid is widely distributed in plant families such as the *Lamiaceae*, *Rosaceae*, and *Myrtaceae*. It exhibits many health-promoting properties, e.g., anticancer, antioxidant, and anti-inflammatory effects [82]. Ursolic acid reduced elevated ALT and AST activities in in vivo experiments. This effect resulted from activation of the FXR, inhibiting CYP7A1 and SREB-1c expression [64]. Additionally, the Nrf2 pathway’s stimulation of ursolic acid may cause this anti-inflammatory action [65,66].

### 7.11. Withaferin A

Withaferin A is a natural steroidal compound derived from *Withania somnifera*, commonly known as Ashwagandha. It is well-recognized for its anti-inflammatory and antioxidant properties, which have led to investigations of its role in liver diseases, including MAFLD [83]. A study by Shirgannavar et al. examined the effects of withaferin A on liver cells and a mouse model of MAFLD [67]. This study demonstrated that withaferin A protects against MAFLD by activating the LXR/FXR receptors. Molecular docking studies confirmed that withaferin A binds effectively to both the FXR and LXR-α, acting as a dual activator of these receptors. The study found that withaferin A restored LXR/FXR receptor activity in animals on a high-fat diet, thereby preventing diet-induced hepatic inflammation and liver fibrosis. Additionally, withaferin A was shown to be a hepatoprotective antioxidant. Research on acetaminophen-induced hepatic toxicity revealed that withaferin’s A protective effects are Nrf2-dependent and mediated through a non-canonical, Keap1-independent mechanism regulated by the phosphatase and tensin homolog (PTEN)/PI3K/Akt axis [68]. Also, research was carried out on the effectiveness of withaferin A on lymphocytes infected with hepatitis C virus [69]. The results demonstrated a significant increase in GSR and GST activities and potential use of withaferin A in HCV.

## 8. Limitation of the Use of Natural Agonists of the FXR and Modulators of Nrf2 in Clinical Practice

Using natural compounds in clinical practice has garnered considerable interest due to their potential health benefits. However, several limitations hinder their widespread adoption in clinical settings. A primary challenge is their low bioavailability, attributed mainly to their high lipophilicity and low solubility in water, resulting in poor absorption from the intestines following oral administration. Among the compounds discussed, lycopene stands out as the most lipophilic. Its long, unsaturated hydrocarbon chain and lack of polar functional groups make it extremely hydrophobic and highly soluble in lipids [84].

In contrast, rutin and hesperidin are glycosylated flavonoids with significant hydrophilicity due to their sugar moieties. While glycosylation typically enhances the bioavailability of flavonoids compared to their aglycone forms, the specific glycosylation type can still limit their absorption. Thus, despite sugar groups, rutin and hesperidin experience less-than-ideal bioavailability [75,85].

The other compounds discussed generally exhibit moderate lipophilicity due to some polar functional groups in their structures but still suffer from low bioavailability. Addressing these bioavailability issues is crucial for translating their therapeutic potential into effective clinical treatments. Several strategies are being explored to enhance bioavailability and maximize efficacy. Nanotechnology is a promising approach involving encapsulating natural compounds in nanoparticles or liposomes to improve their solubility and facilitate targeted delivery. For example, encapsulating lycopene in nanostructured lipid carriers (NLCs) enhances its solubility and stability, improving its bioavailability and therapeutic efficacy. Riangjanapatee et al. [86] demonstrated that incorporating lycopene into NLCs protects this compound from degradation, further improving its stability.

Chemical modifications of the natural compounds, such as esterification or derivatization, can also improve solubility and permeability. For instance, enzymatic hydrolysis has been employed to convert rutin (quercetin-3-O-rutinoside) to quercetin-3-O-glucoside by removing the terminal rhamnose with specific enzymes, resulting in increased bioavailability and higher blood levels of the absorbed compound [85]. Additionally, co-administration with bioavailability enhancers like piperine can improve compound uptake. Wdowiak et al. [87] successfully utilized hot-melt extrusion to produce amorphous systems of curcumin and piperine, significantly increasing their dissolution rate, apparent solubility, permeability, and biological activities. Advanced delivery systems, including sustained-release formulations and transmucosal delivery, also address rapid metabolism and systemic elimination challenges. These approaches collectively enhance the effectiveness and clinical application of natural compounds, paving the way for more widespread use in therapeutic settings. They also highlight promising directions for future research on the discussed compounds, opening avenues for further exploration into optimizing their bioavailability and therapeutic potential. By continuing to refine these strategies, researchers can better harness the full benefits of natural agonists of the FXR and modulators of Nrf2, potentially leading to new treatments for hepatic inflammatory diseases.

## 9. Conclusions

Liver inflammation, a prevalent cause of hepatic diseases, is intricately linked to oxidative stress and dysregulated bile acid metabolism. This review highlighted the interplay between the farnesoid X receptor (FXR) and nuclear factor erythroid 2-related factor 2 (Nrf2) pathways in managing liver inflammation and oxidative damage. The natural compounds explored, including auraptene, curcumin, and resveratrol, demonstrate significant potential in modulating both the FXR and Nrf2 pathways, offering promising therapeutic strategies for liver conditions such as metabolic-associated fatty liver disease (MAFLD) and cholestatic liver injury. Despite their promising efficacy in preclinical models, challenges such as bioavailability and targeted delivery hinder their clinical application. Addressing these limitations through advanced formulations, such as nanoparticle encapsulation, may enhance the therapeutic potential of these natural compounds. Further studies are necessary to explore these strategies and confirm the clinical viability of using natural FXR agonists and Nrf2 modulators to combat hepatic inflammatory diseases.

A promising research prospect seems to be the combination of natural compounds modulating the FXR and Nrf2 pathways with currently used drugs. Demonstrating synergy between established therapies and natural compounds may lead to more effective treatment regimens, potentially reducing side effects, and improving patient outcomes.

## Figures and Tables

**Table 1:** The therapeutic potential of natural FXR and Nrf2 activators in inflammatory liver diseases.

Natural Compounds /Origin	Chemical Name	Model/Efficient Dose	Activation of FXR or Nrf2 Pathway	References
Auraptene *Citrus aurantium*	7-{[(2E)-3,7-dimethylocta-2,6-dien-1-yl]oxy}-2H-1-benzopyran-2-one (purity > 98%)	Model: cholestatic liver injury In vitro: mouse primary cultured hepatocytes (5, 10 and 20 μM) In vivo: C57BL/6 mice (7.5, 15, 30 mg/kg)	↓ ALT, AST, ALP, TBIL, and TBA serum levels ↑ BSEP, MRP2 mRNA, and protein levels ↓ NTCP mRNA and protein level ↓ CYP7A1 and CYP8B1 mRNA levels ↑ SULT2A1 and SHP mRNA levels ↑ FXR protein level	[49]
		In vitro: HepG2 cells (25, 50, 75 and 100 µM) In vivo: ICR mice (150 mg/kg)	↑ GST activities	[50]
Cafestol *Coffea arabica*	3,18-(epoxymetheno)-19-nor-5β, 8α, 9β, 10α, 13β, 16β-kaur-3-ene-16α, 17-diol (purity > 98%)	In vivo: HepG2 cells (56 μM) and CV-1 cells (1, 10, 20 μM) In vivo: C57BL6 mice (400 mg/kg)	↓ CYP7A1, CYP8B1, and NTCP mRNA levels ↑ SHP and BSEP mRNA levels	[51]
		Model: carbon tetrachloride-induced liver damage In vivo: ICR mice (10–100 mg/kg)	↓ ALT and AST in serum levels ↑ GST activities	[52]
Curcumin *Curcuma longa*	(1E,6E)-1,7-bis(4-hydroxy-3-methoxyphenyl)hepta-1,6-diene-3,5-dione (purity > 98%)	Model: MAFLD In vitro: primary hepatocytes from the liver of C57BL/6 mice (10 µM) In vivo: C57BL/6 mice (50, 100 mg/kg)	↓ TG, TC, and ALT serum levels ↑ CYP3A and CYP7A protein level ↓ SREBP-1c protein and mRNA level, FAS mRNA level ↑ FXR, SHP, and Nrf2 protein levels	[53]
		Model: ethanol-induced hepatic steatosis In vitro: Human LO2 hepatocyte (10, 20, 40 µM) In vivo: Sprague-Dawley Rats (100, 200, 400 mg/kg)	↓ AST, ALT, ALP, and LDH serum levels ↓ SREBP-1c and FAS protein and mRNA levels ↑ FXR, Nrf2 mRNA protein, and mRNA levels	[3]
Fargesone A *Magnolia fargesii*	(2S,3R,3aR,7S,7aS)-2-(1,3-benzodioxol-5-yl)-3a,4-dimethoxy- 3-methyl-7-prop-2-enyl-2,3,7,7a-tetrahydro-1-benzofuran-6 (purity-no data)	Model: oleic acid-induced lipid accumulation/bile duct ligation in mice In vivo: WRL68 cells (10 µM) In vivo: C57BL/6 mice (3 mg/kg, 30 mg/kg)	↑ SHP and BSEP mRNA levels ↓ CYP7A1 and CYP8B1 mRNA levels	[54]
Hesperidin *Citrus aurantium*	(2S)-5-hydroxy-2-(3-hydroxy-4-methoxyphenyl)-7- [(2S,3R,4S,5S,6R)-3,4,5-trihydroxy-6-[[(2R,3R,4R,5R,6S)-3,4,5-trihydroxy-6-methyloxan-2-yl]oxymethyl] oxan-2-yl]oxy- 2,3-dihydrochromen -4-one (purity > 97%)	Model: ANIT-induced cholestatic In vitro: HepaRG cells (6.25, 12.5, 25 and 50 µM) In vivo: C57BL/6J mice (25, 50, 100 mg/kg)	↓ ALT, AST, ALP, γ-GT, and TBIL serum levels ↑ FXR, SHP, OATP1A1, BSEP, and MRP2 mRNA levels ↓ NTCP, CYP7A1, and CYP27A1 mRNA levels	[55]
		Model: MAFLD In vitro: HepG2 cells (2,5; 5 and 10 µM) In vivo: Wistar rats (100, 300 mg/kg)	↑ SOD, GPx, GCLC, and HO-1 protein levels ↑ Nrf2 and p-Nrf2 protein levels ↑ Nrf2-Keap1 complex dissociation ↑ Nrf2 translocation to the nucleus	[56]
Lycopene *Solanum lycopersicum*	(6*E*,8*E*,10*E*,12*E*,14*E*,16*E*,18*E*,20*E*,22*E*,24*E*,26*E*)-2,6,10,14,19,23,27,31-octamethyldotriaconta-2,6,8,10,12,14,16,18,20,22,24,26,30-tridecaene (purity > 98%)	Model: chlorpyrifos- induced hepatic toxicity In vivo: Wistar rats (5, 10 mg/kg)	↓ ALT, AST, ALP, and LDH serum levels ↑ SOD, GSH, and GST activities ↑ Nrf2 protein level ↑ HO-1 mRNA level	[57]
		Model: aflatoxin B1- induced hepatic toxicity In vivo: kunming mice (5 mg/kg)	↓ ALT and AST serum activities ↑ SOD, CAT, GSH, and GST activities ↑ Nrf2 protein level ↑ NQO1, SOD1, and CAT mRNA levels	[58]
Oleanolic acid *Olea europaea*	(4aS,6aR,6aS,6bR,8aR,10S,12aR,14bS)-10-hydroxy-2,2,6a,6b,9,9,12a-heptamethyl-1,3,4,5,6,6a,7,8,8a,10,11,12,13,14b-tetradecahydropicene-4a-carboxylic acid (purity > 97%)	Model: ANIT-induced cholestasis In vitro: Human LO2 hepatocyte (20 µM) In vivo: Sprague-Dawley rats (100 mg/kg)	↓ ALT, AST, ALP, TBIL, γ-GT, and TBA serum levels ↑ SOD and GSH activities ↑ Nrf2, FXR, BSEP, and UGT1A1 mRNA and protein levels ↓ CYP7A1 mRNA levels ↑ Nrf2-Keap1 complex dissociation	[40]
Resveratrol *Vitis vinifera*	5-[(E)-2-(4-hydroxyphenyl)ethen-1-yl]benzene-1,3-diol (purity > 98%)	Model: MAFLD In vivo: Wistar rats (25 mg/kg)	↓ AST, ALT, and ALP serum activity ↑ FXR and SIRT1 mRNA levels	[59]
		Model: ANIT-induced cholestasis In vitro: HEK293 cells, HepG2 cells, primary mouse hepatocytes (3, 10, 30 µM) In vivo: WT C57/BL mice (60 mg/kg)	↓ ALT, AST, ALP, TBIL, DBIL, and TBA serum levels ↑ BSEP and SHP mRNA levels ↓ OSTß, MRP3, and MRP2 mRNA levels ↓ CYP7A1 and CYP8B1 mRNA levels	[60]
		Model: MAFLD In vitro: HepG2 cells (20 µM) In vivo: C57/BL6 mice (0.4 % resveratrol)	↓ SREBP-1c and FAS mRNA levels ↓ Keap1 mRNA and protein level ↑ Nrf2 mRNA and protein level ↑ HO-1, NQO1, CAT, and SOD2 mRNA levels ↓ Nrf2 promoter methylation	[61]
Rutin *Fagopyrum esculentum*	2-(3,4-dihydroxyphenyl)-5,7-dihydroxy-3-[(2*S*,3*R*,4*S*,5*S*,6*R*)-3,4,5-trihydroxy-6-[[(2*R*,3*R*,4*R*,5*R*,6*S*)-3,4,5-trihydroxy-6-methyloxan-2-yl] oxymethyl]oxan-2-yl] oxychromen-4-one (purity > 98%)	Model: BDL-induced liver injury In vivo: Sprague-Dawley rats (25 mg/kg)	↓ AST, ALT, TBIL, TG, and TC serum levels ↑ Nrf2 and HO-1 protein levels ↑ p-AMPK protein level ↑ liver CAT and SOD1 activities ↑ GSH protein level	[62]
		Model: diabetic MAFLD In vitro: HeLa cells (150 µM), HepG2 cells (20 µM) In vivo: C57BLKs mice (100 mg/kg, 200 mg/kg)	↓ ALT, AST, TG, and TC serum levels ↑ Nrf2, HO-1, and NQO1 protein levels ↓ FAS, SREBP1-1c, and SCD1 protein levels ↑ p-AMPK protein level	[63]
Ursolic acid *Malus domestica*	(1S,2R,4aS,6aR,6aS,6bR,8aR,10S,12aR,14bS)-10-hydroxy-1,2,6a,6b,9,9,12a-heptamethyl-2,3,4,5,6,6a,7,8,8a,10,11,12,13,14b-tetradecahydro-1H-picene-4a-carboxylic acid (purity > 93%)	Model: ethanol-induced hepatic injury In vivo: rats (150 mg/kg)	↓ ALT, AST, ALP, and TBA serum levels ↓ SREBP-1c and CYP7A1 mRNA levels ↑ FXR protein level	[64]
		Model: carbon tetrachloride-induced liver damage In vivo: ICR mice (25 mg/kg, 50 mg/kg)	↓ AST and ALT in serum activity ↑ SOD, CAT, and GPx activities ↑ Nrf2, NQO1, GST, and HO-1 protein levels	[65]
		Model: ANIT-induced cholestasis In vitro: HepG2 cells (32 µM) In vivo: Sprague-Dawley rats (10 mg/kg, 20 mg/kg, 40 mg/kg)	↓ ALT, AST, ALP, γ-GT, TBIL, DBIL, and TBA serum levels ↑ Nrf2, BSEP, and MRP2 mRNA levels	[66]
Withaferin A *Withania somnifera*	(1S,2R,6S,7R,9R,11S,12S,15R,16S)-6-hydroxy-15-[(1S)-1-[(2R)-5-(hydroxymthyl)-4-methyl-6-oxo-2,3-dihydropyran-2-yl]ethyl]- 2,16-dimethyl-8-oxape tacyclo [9.7.0.02,7.07,9.012,16] octadec-4-en-3-one (purity > 79%)	Model: induced MAFLD In vitro: HepG2 and Huh7 cells (1, 2.5, 5 µM) In vivo: Swill albino mice (1.25 mg/kg)	↓ AST, ALT, and ALP serum levels ↓ TG and TC serum levels	[67]
		Model: acetaminophen-induced hepatic toxicity In vitro: mouse embryonic fibroblasts (MEFs) (0–3 µM) In vivo: C57BL/6J mice (7 mg/kg)	↓ ALT serum level ↑ NQO1, GCLC, GSTP1, UGT1A1, GSTA1, GST1, and HO-1 mRNA levels ↑ Nrf2 protein and mRNA levels	[68]
		Model: lymphocyte cell infection with HCV serum In vitro: human lymphocyte from normal cells (25 mg/mL, 50 mg/mL)	↑ GSR activity ↑ GST activity	[69]

↑/↓ increase/decrease. Abbreviations: ALP, alkaline phosphatase; ALT, alanine aminotransferase; AMPK, AMP-activated protein kinase; ANIT, α-naphthyl isothiocyanate; AST, aspartate aminotransferase; BDL, bile duct ligation; BSEP, bile salt export pump; CAT, catalase; CYP27A1, sterol 27-hydroxylase; CYP3A, cytochrome P450 3A; CYP7A1, cholesterol 7 alpha-hydroxylase; CYP8B1, sterol 12- alpha-hydroxylase; DBIL, direct bilirubin; FAS, fatty acid synthase; FXR, farnesoid X receptor; GCLC, glutamate cysteine ligase catalytic subunit; GPx, glutathione peroxidase; GSH, glutathione; GST, glutathione S-transferases; GSTA1, glutathione S-transferase alpha 1; GSTP1, glutathione S-transferase P; GSR1, glutathione reductase 1; HCV, hepatitis C virus; HO-1, heme oxygenase-1; Keap1, Kelch-like ECH-associated protein 1; LDH, lactate dehydrogenase; MRP2, multidrug resistance-associated protein 2; MRP3, multidrug resistance-associated protein 3; MRP4, multidrug resistance-associated protein 4; MAFLD, metabolic-associated fatty liver disease; NQO1, NAD(P)H oxidoreductase 1; Nrf2, nuclear factor erythroid 2-related factor 2; NTCP, Na+-taurocholate transporting polypeptide; OATP1A1, organic anion transporter polypeptide 1a1; OSTß, organic solute transporter beta; p-AMPK, phosphorylated AMPK; p-Nrf2, phosphorylated Nrf2; SCD1, stearoyl-CoA desaturase; SHP, small heterodimer partner; SIRT1, sirtuin 1; SOD, superoxide dismutase; SOD1, copper–zinc superoxide dismutase; SOD2, manganese-dependent superoxide dismutase; SREBP-1c, sterol regulatory element-binding transcription factor 1c; SULT2A1, sulfotransferases 2a1; TBA, total bile acid; TBIL, total bilirubin level; TC, total cholesterol; TG, triacylglyceride; UGT1A1, UDP-glucuronyl transferase 1A1; γ-GT, gamma-glutamyl transferase.
